# Hesperidin methyl chalcone alleviates imiquimod-induced psoriasis in mice: effects alone and in combination with methotrexate

**DOI:** 10.1007/s00210-025-04811-7

**Published:** 2025-12-12

**Authors:** Hagar W. Abdelrahman, Shadia M. Kadry, Wafaa A. Morsy, Marwa T. Hassen

**Affiliations:** Zoology Department, Faculty of Women for Arts, Science and Education, Ain Shams University, Cairo, 11757 Egypt

**Keywords:** Plaque psoriasis, Hesperidin methyl chalcone, Methotrexate, Imiquimod

## Abstract

Psoriasis is a common chronic inflammatory skin disease with an increasing worldwide prevalence. Hesperidin methyl chalcone (HMC), a water-soluble derivative of the flavonoid “hesperidin”, exhibits antioxidant and anti-inflammatory effects. However, its effects on psoriasis have never been investigated. Therefore, this study aims to evaluate the ameliorative effects of HMC, alone and in combination with methotrexate (MTX) in an imiquimod (IMQ)-induced psoriasis mice model. Twenty-five adult female BALB/c mice were randomized into five groups. Except for the control group, all mice received topical IMQ cream (62.5 mg of 5%) for six consecutive days; controls received Vaseline instead as a vehicle. Treated groups were orally administered MTX (1 mg/kg body weight “b.wt”), HMC (500 mg/kg b.wt), or their combination once daily. Our data revealed that HMC alone markedly attenuated skin erythema, scaling, epidermal hyperplasia, body weight loss, and histopathological alterations, while significantly (*P* < 0.001) suppressed splenomegaly and oxidative stress, and reduced the elevated levels of interleukin (IL)-23, IL-17A, and cyclooxygenase-2, as well as downregulated the expression of tumor necrosis factor-α. Importantly, the combination of HMC with MTX demonstrated a significant superior efficacy compared to either agent alone, suggesting potential additive or synergistic benefits. In conclusion, this study provides the first preclinical evidence that HMC, particularly in combination with MTX, ameliorates IMQ-induced psoriasis via modulation of inflammatory cytokines, oxidative stress, and histopathological damage. These findings suggest its potential as a therapeutic candidate that warrants further preclinical and clinical investigation in plaque-type psoriasis.

## Introduction

Psoriasis is a chronic T cell-mediated autoimmune skin disease (Griffiths et al. 2021; Zhou et al. 2022). The psoriasis prevalence is rising and affects approximately 125 million people globally (Armstrong and Read 2020; Iskandar et al. 2021). Plaque psoriasis is a relapsing and the most prevalent form of psoriasis, which affects more than 80% of psoriasis patients (Walter 2022). It is characterized by hyperproliferation of the epidermal cells (acanthosis), leukocytes infiltration, and erythematous skin with silvery scales on the skin, which accumulate and appear symmetrically most often on the scalp, knees, elbows, and lumbar area on the skin (Branisteanu et al. 2022; Zhou et al. 2022). The etiology of this disease remains complicated and unclear, but there are many risk factors of psoriasis, which include interaction between genetics, environment, and disturbances in the innate and adaptive immune responses to trigger the development of psoriasis (Yamanaka et al. 2021; Branisteanu et al. 2022; Dhabale and Nagpure 2022; Wu et al. 2023b). Psoriasis leads to adverse effects on patients and society and causes many comorbidities, such as increasing risk factors with psoriatic arthritis, depression, cardiometabolic syndrome, and sleep disorders. This critical effect on the quality of life of psoriatic patients can lead to suicidality (Armstrong and Read 2020; Hedemann et al. 2022; Jiang et al. 2023).

Aldara cream, which includes 5% imiquimod (IMQ) is an agonist for toll-like receptors (TLRs)−7/8, and it has been approved for the treatment of perianal and genital warts (van der Fits et al. 2009). The topical IMQ administration induces psoriasis-like skin inflammation in mouse models that resembles psoriasis in humans via activating the interleukin (IL)−23/IL-17A axis, leading to keratinocyte proliferation and secretion of pro-inflammatory cytokines (Luo et al. 2020; Abreu et al. 2023; Sawa et al. 2023; Alfardan et al. 2024). Therefore, IMQ-induced mouse inflammatory model is commonly used to evaluate treatments for persistent psoriasis (van der Fits et al. 2009; Abbas et al. 2025).

There are a variety of therapies available for treating psoriasis, including topical therapies (corticosteroids, vitamin D3 analogs, salicylic acid, and retinoids), oral medication (methotrexate “MTX”, acitretin, and cyclosporine), biological agents (such as the tumor necrosis factor “TNF” inhibitors: etanercept, adalimumab and infliximab, as well as the IL-12/23 inhibitor: ustekinumab), and ultraviolet phototherapy, but they all have financial burdens and severe side effects (Salman et al. 2024; Kaye et al. 2025; Ridha-Salman et al. 2025). Among them MTX (4-amino-10-methyl folic acid) is the first-line and low-priced therapy for moderate to severe psoriasis, but its use is limited by adverse effects such as hepatotoxicity, nephrotoxicity, salivary gland impairment, testicular damage, and bone marrow toxicity (Abdelhameed et al. 2022; Singh et al. 2022). Hence, increasing attention has been directed toward developing chemically modified herbal and natural compounds as safer and more effective anti-psoriatic agents, either alone or in combination with MTX, to improve efficacy and reduce toxicity (Mazhar et al. 2023; Fan et al. 2024).

The medicinal herbs and flavonoids have beneficial palliative effects on psoriasis and many disorders due to their anti-inflammatory and antioxidant activities (Ebrahimi et al. 2024).

Hesperidin is a flavonoid from the Flavanone family and presents in citrus fruits and tangerine peels (Pyrzynska 2022; Buzdagli et al. 2023). It acts as an antioxidant and anti-inflammatory effects via reduction of inflammatory cytokine IL-17, neutrophil recruitment, and oxidative stress (Li et al. 2019; Ji et al. 2024). Hesperidin showed a remarkable improvement in IMQ-treated male BALB/c mice compared with methotrexate (MTX), highlighting its potential as an effective treatment for plaque psoriasis (Li et al. 2019). Hesperidin similar to many other flavonoids has a low water solubility and is poorly absorbed by the intestine (Gil-Izquierdo et al. 2001; Kuntic et al. 2014; Avila-Galvez et al. 2021). On the contrary, hesperidin methyl chalcone (HMC; C_29_H_36_O_15_), a product of methylation of hesperidin under alkaline conditions is made up of a combination of chalcone and flavanone that is more water soluble than hesperidin alone and improves intestinal absorption, bioavailability, and tissue distribution (Bussmann et al. 2022; Borghi et al. 2023).

HMC is safe and exhibits strong antioxidant and anti-inflammatory properties by inhibiting pro-inflammatory cytokine production, limiting neutrophil recruitment, and reducing oxidative stress (Bussmann et al. 2022; Artero et al. 2023). These effects have led to its wide use in mouse models of acute and chronic inflammation and pain, highlighting its potential as a safe multi-target therapeutic agent for immune-mediated diseases such as arthritis, while also protecting against UVB-induced skin damage (Martinez et al. 2016; Ruiz-Miyazawa et al. 2018). Furthermore, a previous clinical trial demonstrated that HMC supplementation at a dose of 500 mg was effective in reducing muscle damage and pain, without causing organ toxicity (Luque et al. 2023). However, to the best of our knowledge, the effects of HMC against IMQ-induced psoriasis have not been studied yet. Thus, this study focused on investigating the protective effects of HMC in preventing psoriasis triggered by IMQ exposure.

## Materials and methods

### Drugs and chemicals

5% IMQ cream was purchased from MEDA (AB, Sweden), HMC was purchased from Super Smart (SA, Luxemburg) as tablets each tablet containing 500 mg HMC (purity ≥ 98%), and MTX was purchased from Techno Pharma for Investment and Development (Giza, Egypt) as tablets, each tablet contains 2.5 mg MTX (purity ≥ 99%). Kits for determining skin tissue content of reduced glutathione (GSH; catalog number: 220307), malondialdehyde (MDA; catalog number: 221203), and superoxide dismutase (SOD; catalog number:220619) were purchased from (Bio-diagnostics Company, Giza, Egypt). All other chemicals used were of analytical grade.

### Animals and ethics statement

Female BALB/c mice (aged 6–8 weeks), weighing 20–25 g, were obtained from the Theodor Bilharz Research Institute's breeding unit (Giza, Egypt). Before the onset of the experiment, mice were acclimated to laboratory settings for a week in clean polypropylene cages in the specific pathogen-free facility, having a temperature-controlled room at 25 ±  2 °C with a humidity level of 35–50% and a 12-h light/dark cycle. The mice had a standard pellet diet (Cairo Feed Company, Giza, Egypt). Following the National Institutes of Health's criteria (NIH publication No. 86–23, revised 1985), the animal handling procedure received approval from the Research Ethics Committee at the Faculty of Women for Arts, Science, and Education, Ain Shams University (Approval code: sci1312307004).

### Experimental design

**Fig. 1:**
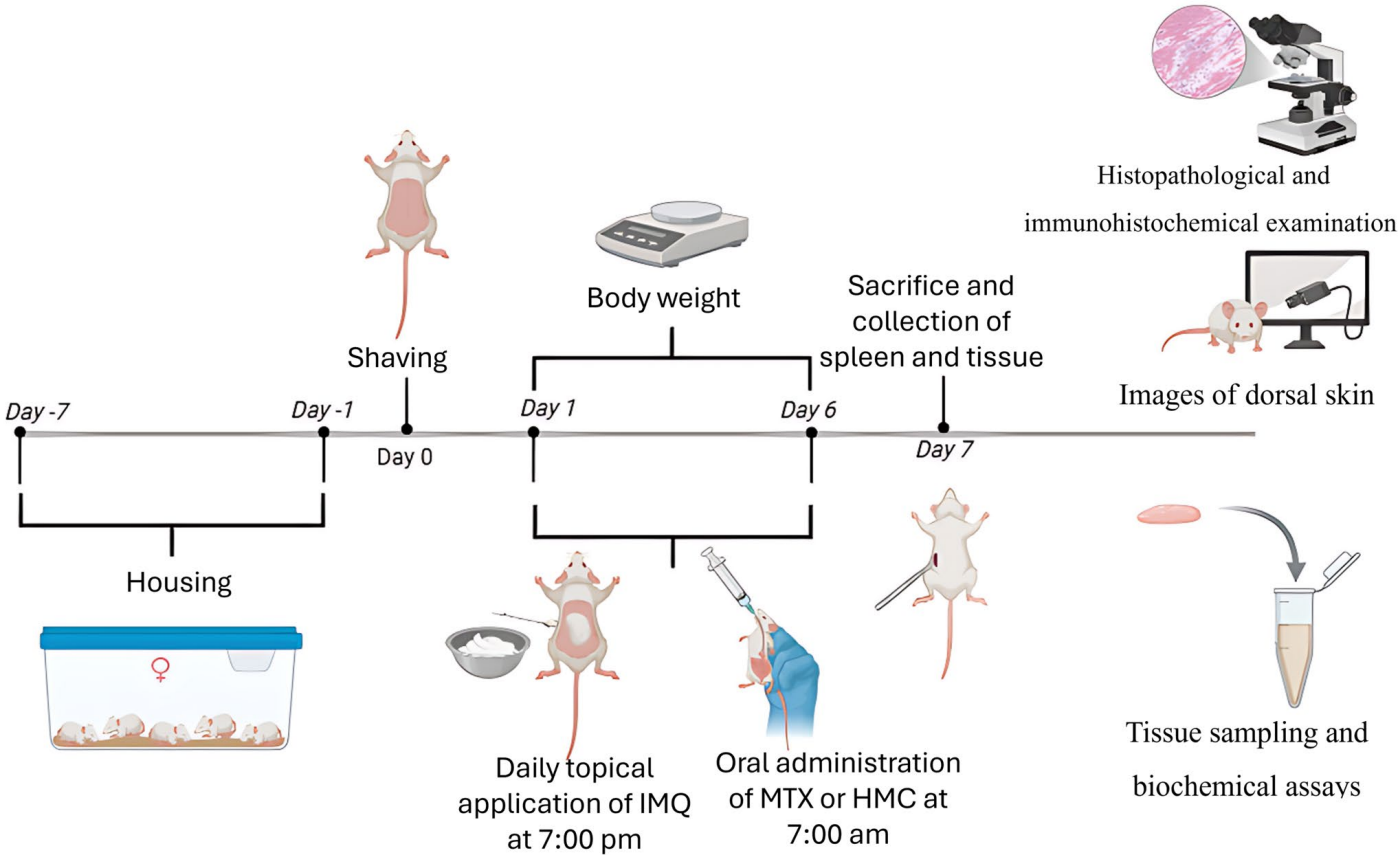
Experimental design of the IMQ-induced psoriatic inflammation mouse model. HMC: hesperidin methyl chalcone; IMQ: imiquimod; MTX: methotrexate

After one week of acclimation, the dorsal skin of all mice was shaved using an electric clipper, followed by complete elimination of any remaining hairs via depilatory cream and randomly divided into five groups: control group, IMQ group, MTX-treated group, HMC-treated group, and MTX + HMC-treated group (n = 5 mice/group). To establish the plaque psoriasis model (Fig. 1), 62.5 mg of IMQ cream was topically administered on the dorsal shaved skin of all mice groups (size 3 × 3 cm^2^) for 6 consecutive days at 7:00 pm, except for the control group, mice were treated with Vaseline. HMC (500 mg/kg body weight “b.w”) and MTX (1 mg/kg b.w) were dissolved in 200 μl distilled water and orally administered via oral gavage to mice in the treated groups once daily at 7:00 a.m., approximately 12 h before applying IMQ on the dorsal skin for 6 consecutive days, while distilled water was administered orally to the control and IMQ groups. The doses were chosen based on a pilot study and previous investigations on IMQ or MTX (van der Fits et al. 2009; Zong et al. 2020) and a previous study on hesperidin and HMC (Li et al. 2019; Luque et al. 2023).

### Assessment of psoriasis-like symptoms

The severity of psoriatic inflammation of the back skin of the experimental mice were scored using the psoriasis area and severity index (PASI) described by (van der Fits et al. 2009; Luo et al. 2016; Wu et al. 2023a; Ali et al. 2025). Mice were evaluated on days (0, 2, 4, and 6) according to skin erythema, scaling, and thickness on a 0–4 scale (0, none; 1, slight; 2, moderate; 3, marked; and 4, sever). Then the PASI was calculated for the three parameters (erythema + scaling + skin thickening) which leading to a cumulative score from 0 to 12. The qualitative assessment was performed by a researcher who was unaware of the identity of the individual samples.

### Body weight and spleen index

Mice were weighed daily until the experiment was over by using a digital balance. Following sacrifice, the spleens were harvested, cleaned out, weighed, and photographed using a camera to evaluate splenomegaly. The formula for calculating the spleen index is as follows (Yang et al. 2022):

Spleen index = spleen weight in grams/body weight in grams × 100.

### Tissue sampling and biochemical assays

On the 7th day, images of the shaved portion of the mice from all experimental groups were captured using a camera. Following the euthanasia of mice by inhalant overdose of isoflurane, shaved back skin was collected and washed with saline (0.9% NaCl) to get rid of any blood clots. Then skin tissue was cut into two parts; one was small pieces (0.2 g), stored at −80°C, and homogenized at 4 °C for 15 min in 2 ml of phosphate buffer solution by a variable speed homogenizer and centrifuged. The resulting filtrate was used for the estimation of antioxidant markers (AbdelKader et al. 2023), including GSH, SOD, and MDA measured by commercial kits, and a microplate reader (Thermo Fisher Scientific, USA) according to the manufacturer’s instructions. The other part of the skin was used for histopathology and immunohistochemistry.

### Estimation of inflammatory markers

The skin tissue homogenate was also analyzed to determine the levels of inflammatory mediators, IL-17A (catalog number: MBS2508197), cyclooxygenase-2 (COX-2; catalog number: MBS269104) were obtained from (MyBiosource Company, San Diego, U.S), and IL-23 (catalog number: E-EL-M0731) was purchased from (Elabscience Company, Houston, U.S). These markers were quantified using commercial ELISA kits, and the absorbance was measured with a microplate reader (Thermo Fisher Scientific, USA) according to the manufacturer’s protocols (Ghahartars et al. 2021; Mardani et al. 2021).

### Histopathological and immunohistochemical examination

After being preserved in 10% neutral-buffered formalin, the dorsal skin was embedded in paraffin. The paraffin slices (5 μm) were stained with haematoxylin and eosin (H&E) staining according to (Wu et al. 2023a). Then the Baker’s scoring system was used to evaluate the degree of histopathological alterations on a scale ranging from 0 to 10. All the sections were scored blindly by an independent researcher, and the mean score was recorded (Luo et al. 2016).

Moreover, other sections were incubated with polyclonal antibodies for TNF-α (catalog no. SC7269, Santa Cruz, CA, USA) at 4 °C overnight, followed by incubation with horseradish peroxidase-conjugated secondary antibody (Ventana, AZ, USA) for 60 min at room temperature. Ultimately, the positive staining was visualized by using a diaminobenzidine kit (Ventana, AZ, USA) and hematoxylin was used to counterstain the nucleic acid. The images were captured and examined at a magnification power of 400 × under an electric light microscope (Olympus U-LH 100HG, Tokyo, Japan). The percent of immunoreactivity area of TNF-α in skin tissues were analyzed and measured with ImageJ Software (National Institutes of Health, Bethesda, MD, United States) as previously recommended (Li et al. 2021).

### Statistical analysis

All qualitative data are presented as mean ± standard error of the mean. Statistical analysis was performed with repeated measures and one-way analysis of variance (ANOVA), and Kruskal–Wallis test followed by Bonferroni’s and Dunn's multiple comparison post-hoc tests. Normality and homogeneity of error variance of dependent variable was tested using Kolmogorov–Smirnov test. All research data were analyzed using GraphPad Prism software version 4.03 for Windows (GraphPad software; San Diego, CA, USA). *P* values of < 0.05, < 0.01, and < 0.001 were regarded as statistically significant, highly significant, and very highly significant, respectively.

## Results

### HMC ameliorated psoriatic symptoms in IMQ-induced mouse model of psoriasis

**Fig. 2:**
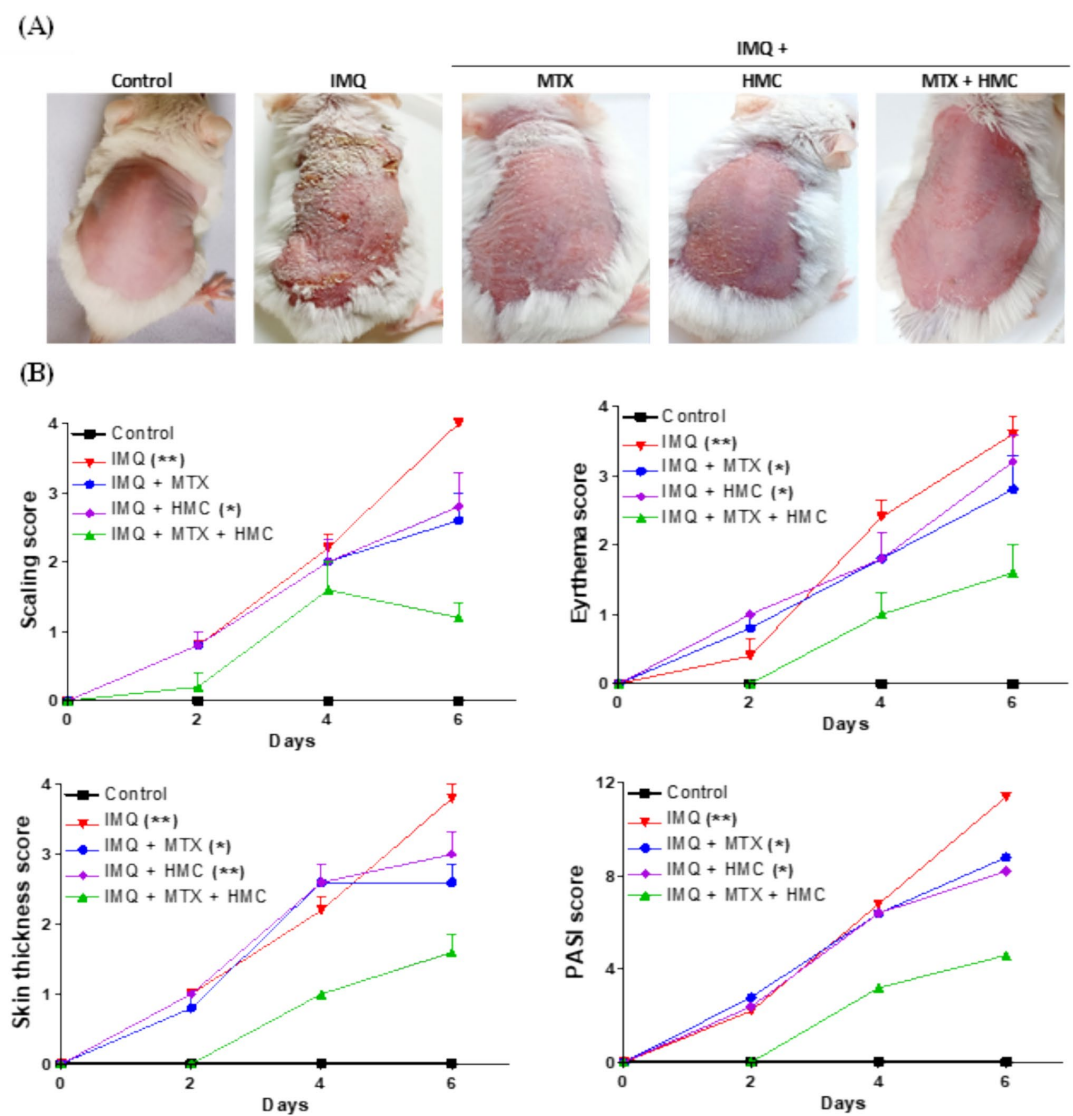
Effect of HMC or/and MTX on the psoriatic severity in IMQ-induced psoriatic skin lesions. **A** Representative photographs of mice dorsal skin on day 7. The control group showed no psoriatic symptoms. In contrast, the IMQ group showed severe psoriatic inflammation in the form of scaling and erythema. While these psoriatic symptoms were markedly decreased in the treated groups. **B** The scores of erythema, scaling, skin thickness, and PASI during the experimental period. HMC: hesperidin methyl chalcone; IMQ: imiquimod; MTX: methotrexate; PASI: psoriasis area and severity index. Data are expressed as means ± their standard errors. n = 5 in each group. * and **: *P* < 0.05 and *P* < 0.01 compared with the control group (Repeated measure ANOVA)

As shown in Fig. 2A, on day 7 and by end of the experimental period, the morphological examination of the skin revealed that the IMQ group appeared obvious erythema and scaling, which are the two major clinical manifestations of psoriasis in comparison to the control group (Vaseline-treated group) which didn’t show any signs of inflammation on the dorsal skin (Fig. 2A). However, oral pre-treatment with MTX or HMC markedly lowered the psoriatic skin features, resulting in smoother skin and fewer scales and erythema, compared to those groups of mice treated with IMQ (Fig. 2A). Furthermore, the combination of both treatments exhibited better anti-psoriatic effect than individual treatments (Fig. 2A), where the complete disappearance of excessive silvery-white scaling and mild recovery from erythema were observed.

This observation was further corroborated by the PASI sore (including 3 indices: erythema, scaling, and skin thickness), which were consistent with the symptoms visible in the images (Fig. 2A and B). These results indicated that the overall performance of the combination of both treatments was superior to that of the other individual treatment groups, which resulted in the most substantial decrease in the cumulative PASI score with no significant differences observed as compared to the control mice (*P* > 0.05, Fig. 2B).

#### HMC attenuated IMQ-induced psoriatic inflammation

**Fig. 3:**
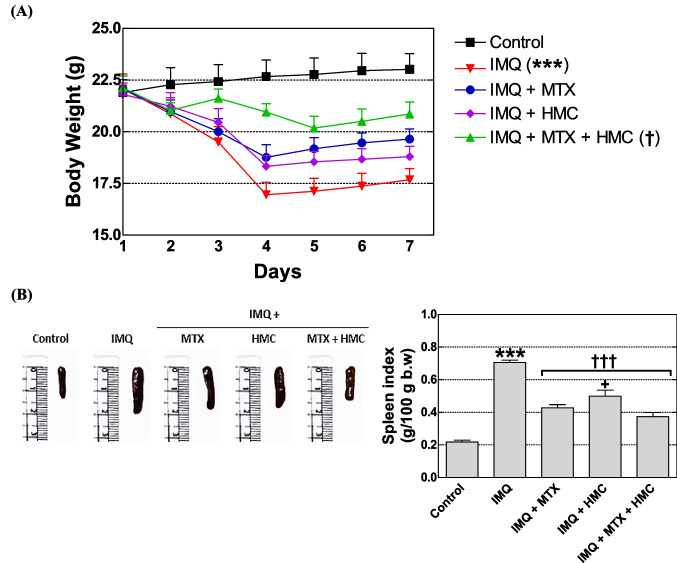
Effect of HMC or/and MTX on splenomegaly and body weight loss of the IMQ-induced psoriasis mouse model. **A** Body weight loss during the experimental period. **B** Representative photographs of the harvested spleen of all groups on day 7 and spleen index. b.w: body weight; HMC: hesperidin methyl chalcone; IMQ: imiquimod; MTX: methotrexate. Data are expressed as means ± their standard errors. n = 5 in each group. ***: *P* < 0.001 compared with the control group; † and †††: *P* < 0.05 and *P* < 0.001 compared with the IMQ group; + : *P* < 0.05 compared with the IMQ + MTX + HMC group (Repeated measure ANOVA for body weight and one-way ANOVA with Bonferroni’s multiple comparison test for spleen index)

The weight loss of mice also represents disease severity (Fig. 3A). Our data indicated that the mice were given IMQ cream on their dorsal skin significantly decreased (*P* < 0.001) their body weight, indicating the diseased state of animals from the 2nd day to the 7th day (Fig. 3A). The animals’ body weight remained unchanged after receiving Vaseline therapy, indicating that there was no negative impact of Vaseline on the animals’ weight (Fig. 3A). However, the animals’ body weight of groups pre-treated with MTX or HMC showed a faint drop on day 2 and gradual increase from the 4th day to the 7th day. Interestingly, this body weight loss was prevented significantly (*P* < 0.05) by the combination of both treatments compared with the IMQ group (Fig. 3A).

The spleen index (spleen weight/100 g b.w) was recorded as an indication of the extent of immune stimulation (i.e., psoriatic inflammation). As shown in Fig. 2, IMQ psoriatic mice exhibited spleen changes compared with control mice. Compared to the control group, the IMQ group had a larger spleen (Fig. 3B), and the spleen index was significantly (*P* < 0.001) higher than the normal spleen of the control group (Fig. 3B), demonstrating immunological activation. After MTX or HMC or the combination treatment for 7 days, all the sizes of spleen tissue as well as the spleen index showed a significant reduction (*P* < 0.001) as compared to the IMQ group (Fig. 3B). Moreover, the combination pre-treatment was more potent (*P* < 0.05) than the HMC in modulating the increase in the spleen index (Fig. 3B).

### HMC improved IMQ-induced depletion of antioxidant defense

**Fig. 4:**
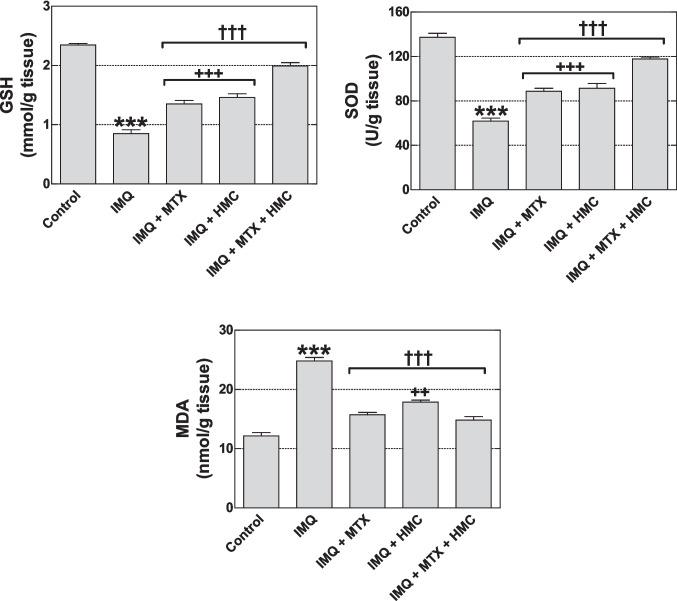
Effect of HMC or/and MTX on skin tissue oxidative stress in the IMQ-induced psoriasis skin lesions. GSH: reduced glutathione; HMC: hesperidin methyl chalcone; IMQ: imiquimod; MDA: malondialdehyde; MTX: methotrexate; SOD: superoxide dismutase. Data are expressed as means ± their standard errors. n = 5 in each group. ***: *P* < 0.001 compared with the control group; †††: *P* < 0.001 compared with the IMQ group; +  + and +  +  + : *P* < 0.01 and *P* < 0.001 compared with the IMQ + MTX + HMC group (One-way ANOVA with Bonferroni’s multiple comparison test)

According to the current investigation, the concentration of GSH and SOD activity in skin tissue was significantly lower (*P* < 0.001) in the IMQ group, whereas MDA level significantly increased (*P* < 0.001), as compared with the control group (Fig. 4). On the other hand, pre-treatment with HMC or/and MTX modulated significantly (*P* < 0.001), the decreasing of the GSH concentration and SOD activity, and the elevation in MDA concentration of the mice exposed to IMQ topical application (Fig. 4). Furthermore, the combination of MTX with HMC was more potent (*P* < 0.01–0.001) than MTX or HMC alone in modulating this skin biochemical alterations of mice exposed to IMQ topical application (Fig. 4).

### HMC downregulated the production of COX-2 and inflammatory cytokines

**Fig. 5:**
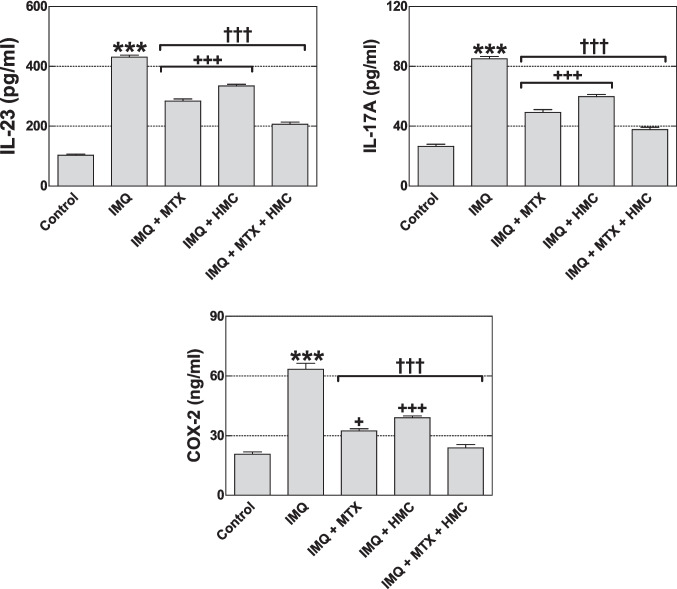
Effect of HMC or/and MTX on skin tissue inflammatory cytokines and COX-2 alterations in the IMQ-induced psoriasis skin lesions. COX-2: cyclooxygenase-2; HMC: hesperidin methyl chalcone; IL: interleukin; IMQ: imiquimod; MTX: methotrexate. Data are expressed as means ± their standard errors. n = 5 in each group. ***: *P* < 0.001 compared with the control group; †††: *P* < 0.001 compared with the IMQ group; + and +  +  + : *P* < 0.05 and *P* < 0.001 compared with the IMQ + MTX + HMC group (One-way ANOVA with Bonferroni’s multiple comparison test)

As shown in Fig. 5, the levels of inflammatory cytokines IL-23, IL-17A, and COX-2 were significantly increased in the skin tissue of the IMQ group (*P* < 0.001) as compared to the control group. However, pre-treatment with MTX or/and HMC showed a significant reduction (*P* < 0.001) in the levels of these inflammatory cytokines and COX-2 as compared to the IMQ group (Fig. 5). Additionally, the combination pre-treatment was more potent (*P* < 0.05–0.001) than the MTX or HMC alone in modulating these alterations of mice exposed to IMQ topical application (Fig. 5).

### HMC alleviated IMQ-induced psoriatic skin lesions

**Fig. 6:**
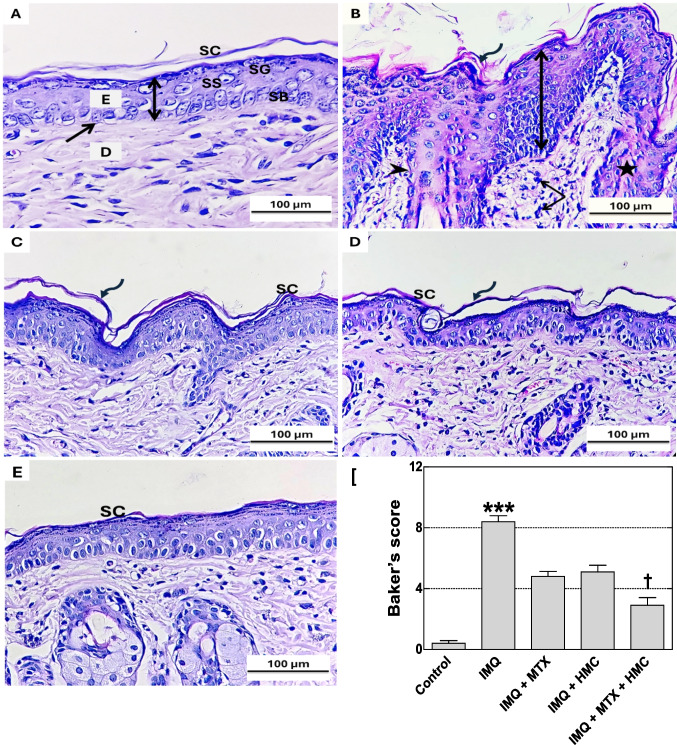
The ameliorative effect of HMC or/and MTX on histopathological alterations in H&E-stained skin section and Backer’s score in psoriatic skin lesions. **A** (control),** B** (IMQ),** C** (IMQ + MTX), **D** (IMQ + HMC), and **E** (IMQ + MTX + HMC) treated groups. The control group showed normal histological architecture of the skin composed of epidermis (E) and dermis (D) and separated by a regular basement membrane (arrow). The epidermis showed regular uniform thickness (double-headed arrow) that was composed of stratum basale (SB), stratum spinosum (SS), stratum granulosum (SG), and stratum corneum (SC). In contrast, the IMQ group showed histopathological changes including acanthosis (double-headed arrow), loosely attached stratum corneum (curved arrow), prominent epidermal rete ridges (star), irregular basement membrane (arrowhead), as well as inflammatory cells infiltration (arrows). While these severe histopathological changes were greatly alleviated in the IMQ groups that were pre-treated with MTX or/and HMC. However, the loosely stratum corneum (SC) is still observed in the group of mice pre-treated with MTX or HMC (curved arrow). Each photograph represents five animals from each group. The scale bar equals 100 µm. **F** (histopathological evaluation based on Baker’s scoring system). H&E: haematoxylin and eosin; HMC: hesperidin methyl chalcone; IMQ: imiquimod; MTX: methotrexate. Data are expressed as means ± their standard errors. ***: *P* < 0.001 compared with the control group; †: *P* < 0.05 compared with the IMQ group (Kruskal–Wallis with Dunn's multiple comparison test)

To confirm the therapeutic effect of HMC against psoriasis caused by IMQ, hematoxylin and eosin (H&E) staining was applied to investigate the histopathological examination of the dorsal skin in mice. Examining dorsal skin slices taken from the control group histologically revealed the skin's typical histological structure in both layers: the epidermis and the dermis, separated by a regular, intact, and flattened basement membrane throughout the sections. The epidermis showed regular uniform thickness that was formed of stratum basale, stratum spinosum, stratum granulosum, and stratum corneum. The cells of the stratum corneum appeared as flat non-nucleated keratinized cells (Fig. 6A). In contrast, the skin sections of the IMQ-treated mice demonstrated a significant increase in Baker’s score (*P* < 0.001, Fig. 6F) with multiple psoriasis-like pathological alterations including an increase in the epidermal thickness (acanthosis), elongation of the rete ridges, and the basement membrane appearing irregular with indistinct boundaries between the dermis and the epidermis, which are the main pathological manifestations of psoriasis, and this was accompanied by infiltration of inflammatory cells (Fig. 6B).

On the other hand, the histopathological examination revealed that pre-treatment with HMC or MTX alleviated these alterations from severe to mild changes, diminished the downward extension of the epidermal rete ridges, reduced the inflammatory cell infiltration and epidermis thickening which were noticed in the IMQ group (Fig. 6C and D). In addition, skin section of the combination group demonstrated a significant (*P* < 0.05, Fig. 6F) amelioration of those severe pathological alterations noticed in the IMQ group and revealed that the MTX and HMC synergistically preserved the skin histoarchitectural integrity nearly normal with clear basement membrane and uniform regular thickness comparable to control group (Fig. 6E).

### HMC alleviated the expression of TNF-α

**Fig. 7:**
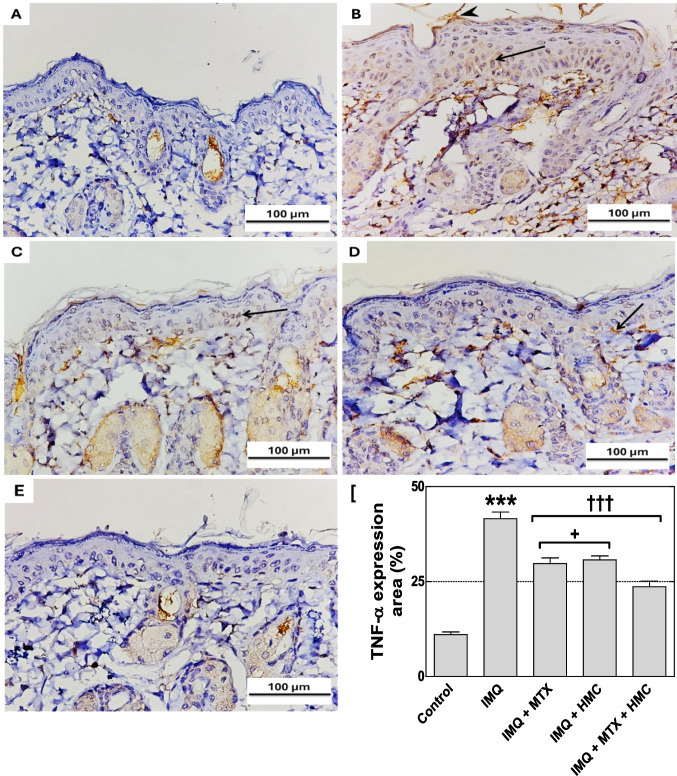
The ameliorative effect of HMC or/and MTX on TNF-α expression in psoriatic skin lesions. **A** (control),** B** (IMQ),** C** (IMQ + MTX), **D** (IMQ + HMC), and **E** (IMQ + MTX + HMC) treated groups. The control group showed negative TNF-α immunoreaction in the epidermis. In contrast, the IMQ group showed a deeply stained brown color of TNF-α immunoreactivity in the keratinocytes' cytoplasm reaching up to the upper layers of the epidermis (arrow) and in the cornea layer (arrowhead). While the groups of mice pre-treated with MTX or HMC showed moderate TNF-α expression (arrow). In addition, the combination group demonstrated faint immunoreactivity for TNF-α in the epidermal region. Each photograph represents five animals from each group. The scale bar equals 100 µm. **F** (percentage area of TNF-α immune expression). HMC: hesperidin methyl chalcone; IMQ: imiquimod; MTX: methotrexate. TNF-α: tumor necrosis factor-α. Data are expressed as means ± their standard errors. ***: *P* < 0.001 compared with the control group; †††: *P* < 0.001 compared with the IMQ group; + : *P* < 0.05 compared with the IMQ + MTX + HMC group (One-way ANOVA with Bonferroni’s multiple comparison test)

As revealed by immunohistochemical staining, the control group had virtually negligible expression of TNF-α (Fig. 7A). In contrast, skin sections of the IMQ-induced group showed a significant upregulation of TNF-α immunoreactivity (*P* < 0.001, Fig. 7F) as evidenced by the strong brown staining in the keratinocyte cytoplasm (Fig. 7B). While this pathological elevation of TNF-α expression was significantly attenuated (*P* < 0.001, Fig. 7F) in the MTX or HMC or combination-treated groups, demonstrating moderate to weak TNF-α reaction (Fig. 7C-E). Moreover, our findings showed that the inhibitory effect of the combination of both treatments on TNF-α expression was more potent than the individual treatments (Fig. 7F).

## Discussion

Psoriasis is a life-long inflammatory skin disorder that can affect the patients’ quality of life (Griffiths et al. 2021; Zhou et al. 2022). Therefore, it is an urgent need to develop novel safe anti-psoriatic compounds, particularly in the absence of an effective medicine with decreased cytotoxicity for long-term use (Liu et al. 2020; Thatikonda et al. 2020; Xu et al. 2021, 2022; Zhang et al. 2021; Khueangchiangkhwang et al. 2023; Yoshida et al. 2023; Ebrahimi et al. 2024). Here, we gave the first evidence that HMC, the chemically modified natural compound, can alleviate the psoriatic inflammation.

It has been demonstrated that IMQ topical administration, a widely used mouse model of psoriasis, can result in psoriasis-like symptoms, including scaling and skin erythema, and this model serves as a suitable tool for discovering therapies for psoriasis (van der Fits et al. 2009; Luo et al. 2020). Erythema and scales on the skin of mice in the IMQ-treated group of the current study may be due to hyperproliferation and aberrant keratinocyte differentiation (parakeratosis) (Liu et al. 2019; Guo et al. 2022; Ortiz-Lopez et al. 2022; Tan et al. 2023). In addition, the present data showed that HMC and MTX alleviated these severe skin inflammation symptoms, and these results were attributed to their anti-inflammatory effects (Zong et al. 2020; Guazelli et al. 2021). Moreover, in the combination group, mice back skin were nearly comparable to the control group.

Along with the typical psoriatic skin lesions, the IMQ-induced psoriatic mouse model exhibited reactions of systemic inflammation, including body weight loss and enlargement of the spleen (van der Fits et al. 2009; Zhang et al. 2020). Also, Zhang et al. (2020) and Zhang et al. (2021) reported that IMQ administration induced weight loss from day 2 to day 7 due to decreased appetite as a result of increasing proinflammatory cytokines and elevation of oxidative stress, which lead to metabolic changes associated with psoriasis. On the other hand, our results showed that the combination of both treatments significantly improved the body weight loss in IMQ-treated mice. Moreover, the involvement of the MTX in increasing body weight may be caused by its effect in lowering the proinflammatory cytokines and reducing infiltration of immune cells (Zhang et al. 2021; Maiti et al. 2023). Similarly, HMC improved the body weight loss that may be due to its ability to reduce oxidative stress, pain, and proinflammatory cytokines (Luque et al. 2023).

Correspondingly, the spleen is a vital immunological organ in the human body that produces a range of immune cells, which play a crucial role in immune response (Elmore 2006). Psoriatic patients often have an active immune system, and their enlarged spleen is an indication of the extent of chronic psoriatic inflammation and the increased number of leukocytes in the spleen, which are then redirected to the site of inflammation (van der Fits et al. 2009; Balato et al. 2015). In the current study, the spleen index of mice in the IMQ group was significantly higher than the normal spleen of the control group, indicating that IMQ can hyperactive the immune system. Besides that, Gangadevi et al. (2021) and Du et al. (2022) noted that the number of immune cells such as T-helper (Th)17/Th22 in the spleen was increased to a high level, which may be a sign of enhanced immunoreaction. Also, Zhang et al. (2021) attributed the increase in spleen index after IMQ administration to the polarization of macrophage 1 as a result of the elevation of IL-17 and TNF-α, as well as activation of T-helper 17, which caused a suppression of macrophage 2. In our study, the MTX administration showed spleen index reduction. This finding was consistent with that of Bedoui et al. (2019) and Zhang et al. (2021), who reported that the MTX was able to inhibit the induction of psoriasis, thus immunoreaction was decreased as a result of MTX acts on macrophage 2 to secrete its anti-inflammation cytokine such as IL-10, as well as it suppresses T-cells and macrophage 1 infiltration in spleen and theirs related pro-inflammatory cytokines such as (TNF-α, IL-6, IL-23). Moreover, HMC pre-treatment decreased the spleen size, indicating the best treatment outcome, which suggested that HMC improved the therapeutic efficacy of MTX. According to these findings, HMC could have an immunomodulatory effect when used to treat psoriasis.

Additionally, many research studies have revealed that oxidative stress is a substantial contributor to psoriasis pathology (Baek et al. 2012; Medovic et al. 2022). More specifically, it has been shown that topical application of IMQ to mouse skin results in an imbalance between oxidants and antioxidants, increased production of reactive oxygen species, and decreased function of antioxidant enzymes, all of which harm dermal and epidermal tissues and cause psoriasis-like skin changes (Baek et al. 2012). Herein, HMC and MTX significantly alleviated psoriasis associated oxidative stress, evidenced by elevated activity of antioxidant enzyme SOD and GSH level, and reduced MDA level in the skin of mice received IMQ. Also, other investigations revealed that the antioxidant properties of HMC and MTX, which reduced tissue lipid peroxidation and restored the depleted levels of GSH and SOD activity, are responsible for their modulatory effects on various inflammatory diseases (Agrawal et al. 2020; Artero et al. 2023). These results are consistent with that of Winiarska-Mieczan et al. (2020), who confirmed the antioxidant effect of MTX in improving the levels of GSH, SOD, and MDA.

The pathogenesis of psoriasis is complicated and remains unclear; however, immunological abnormalities play a crucial role (Medovic et al. 2022). The inflammatory axis mediated by IL-23/IL-17A is crucial in psoriasis pathogenesis (van der Fits et al. 2009; Medovic et al. 2022; Sieminska et al. 2024). The production of TNF-α by immune cells triggers the production of IL-12 and IL-23 by myeloid dendritic cells, ultimately leading to the differentiation of IL-17A producing cells and the production of other inflammatory factors by keratinocytes. The synthesis of several proinflammatory cytokines is triggered by IL-17A and other inflammatory factors acting on keratinocytes, which further leads to a direct influx of immune cells to the site of IMQ administration and amplifies skin inflammation (Sieminska et al. 2024). Therefore, therapeutics that specifically target the IL-23/IL-17A pathway might be a promising strategy for treating psoriasis (Guo et al. 2023). Our data confirmed that the concentration and expression of the main inflammatory factors (IL-23, IL-17A, and TNF-α) were significantly down-regulated in psoriatic mice's skin lesions following pre-treatment with HMC or/and MTX, that most likely because of their anti-inflammatory activity and anti-psoriatic effect (Rasquel-Oliveira et al. 2020; Wang and Jin 2023; Fan et al. 2024). In addition, Li et al. (2019) reported that the potential anti-psoriatic activity of MTX and hesperidin may be attributed to the suppression of immune cells that produce the key cytokines (e.g., IL-23, IL-17A, and TNF-α) driving the inflammatory cascade associated with psoriasis. Therefore, we deduced that HMC could affect the IL-23/IL-17A axis by blocking TNF-α and subsequently indirectly reducing the production of IL-23 and IL-17A.

One step further, the downstream response of pro-inflammatory cytokine COX-2, which is known to be involved in the development and exacerbation of IMQ-induced psoriatic inflammation was examined (Wronski and Wojcik 2022). In this study, the psoriatic lesion exhibits a significant increase in COX-2 levels that may be linked to increased lipid peroxidation accumulation in macrophages, which raises reactive oxygen species and impacts keratinocytes, ultimately causing skin proliferation (Wronski and Wojcik 2022). Of note, HMC or/and MTX pre-treatment significantly inhibited this increase after IMQ application due to their anti-inflammatory effects. These data run in parallel with those obtained by Mello et al. (2000) and Sakata et al. (2003), who suggested that the anti-inflammatory effect of MTX and hesperidin may be due to the reduction of the levels of pro-inflammatory mediators associated with COX-2, such as prostaglandins, thus helping to alleviate inflammation. Also, other studies postulated that the anti-inflammatory effect of hesperidin may be due to its ability to interfere with the COX-2/prostaglandin E_2_ pathway (Siddiqi et al. 2018; Rahmani et al. 2023).

The histological examination also afforded critical support for the biochemical results, which revealed epidermal thickening (acanthosis), elongated rete ridges, and thickening of stratum corneum (hyperkeratosis) after IMQ administration (Guo et al. 2022). Consistent with these results, Rajguru et al. (2020) attributed the increase in epidermal thickness was due to the hyperplasia of keratinocytes. Also, other studies postulated that dysfunctional apoptosis plays an important role in the development of several skin diseases, such as psoriasis, because it is associated with decreased apoptosis along with epidermal hyperplasia or hyperkeratosis (Thatikonda et al. 2020).

In the current work, thickened stratum corneum with many wide spaces between the irregularly organized and loosely attached or almost separated from the underlying keratinocytes was observed after IMQ administration, which may be due to a reduction in lipid composition of stratum corneum (Knox and O'Boyle 2021; Yang et al. 2021). According to Ortiz-Lopez et al. (2022) and Goto et al. (2022), IMQ administration caused acanthosis which may be due to a rapid proliferating capacity with increased mitosis. In addition, excessive inflammation was also observed after IMQ administration in the current study, which may be attributed to the presence of congested and dilated blood vessels in the dermis as well as high levels of proinflammatory cytokines production with the abundant infiltrates of inflammatory cells as a result of releasing TNF-α (Rapalli et al. 2020; Yoshida et al. 2023).

Additionally, as demonstrated by the H&E studies, HMC or/and MTX pre-treatment reversed these histological and pathological changes of psoriasis that were seen in the IMQ-induced psoriasis group. Moreover, the therapeutic effect of HMC was equivalent to that of MTX, signifying that HMC may be effective against psoriasis. This comes in line with Zong et al. (2020); Li et al. (2019), who reported an improvement in skin inflammation symptoms (erythema and scaling) and histopathological changes in MTX or/and hesperidin-treated mice. Thus, they proposed that interference with DNA synthesis and apoptosis of hyperproliferative cells following MTX or/and hesperidin treatment induces a subsequent inhibition of keratinocyte proliferation. Furthermore, our data showed nearly normal improvement in the inflammation of skin tissue after treatment of the IMQ group with HMC, which may be attributed to the ability of HMC to regenerate skin damage and its antioxidant and anti-inflammatory properties in inhibiting neutrophil infiltration (Artero et al. 2023). This was confirmed by the improvement in the high level of COX-2, IL-23, and IL-17A.

**Fig. 8:**
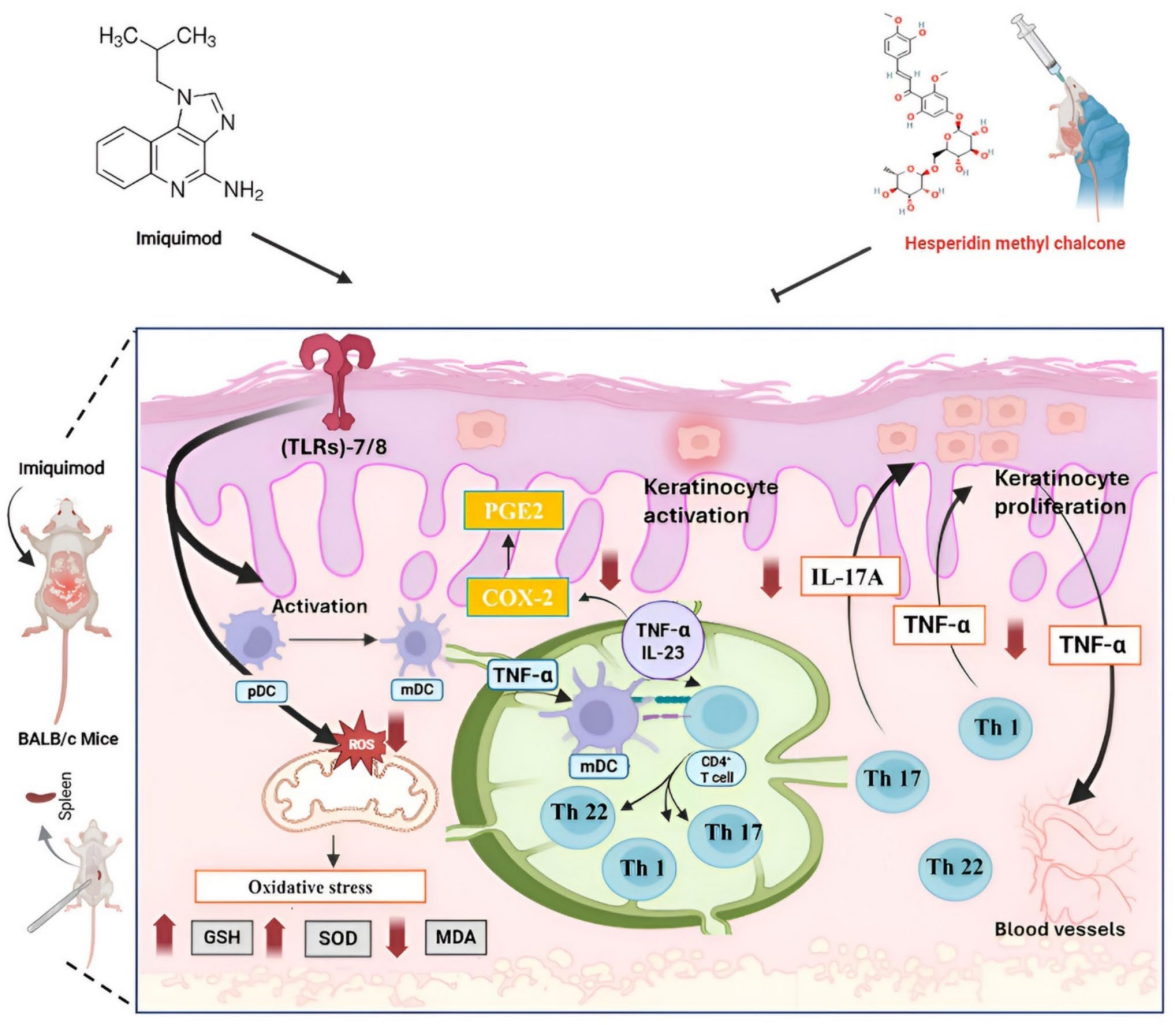
A schematic representation of the modulatory effects HMC (red arrows) on IMQ-induced psoriasis-like dermatitis in mice. Oral HMC pre-treatment demonstrated a substantial reduction in oxidative stress indicators like MDA, and a considerable rise in antioxidant defense like GSH and SOD. It also exhibited dramatically lower skin levels of inflammation-related biomarkers like COX-2, TNF-α, IL-23, and IL-17A. COX-2: cyclooxygenase-2; GSH: reduced glutathione; HMC: hesperidin methyl chalcone; IL: interleukin; IMQ: imiquimod; MDA: malondialdehyde; mDCs: myeloid dendritic cells; pDCs: plasmacytoid dendritic cells; PGE2: prostaglandin E_2_; ROS: reactive oxygen species SOD: superoxide dismutase; Th: T helper; TLRs: toll-like receptors; TNF-α: tumor necrosis factor-α

All of these aspects and the results presented in this study contributed synergistically to offer novel evidence that HMC may assist in controlling psoriatic inflammation by minimizing the severity of psoriatic plaques, reducing splenomegaly, correcting histological alterations, and diminishing the production of oxidative and inflammatory parameters, as seen in Fig. 8.

## Conclusion

Our data provided evidence for the therapeutic potential of HMC (500 mg/kg b.wt) alone and in combination with MTX (1 mg/kg b.wt); the best results are obtained when they are used together. This may be due to their significant antioxidant, anti-inflammatory, and antiproliferative effects. Therefore, our study demonstrates that HMC is effective in improving psoriatic lesions; alleviating tissue levels of oxidative stress, TNF-α, and COX-2, and subsequently blocking the IL-23/IL-17A axis, supporting further investigation of HMC as a potential adjunctive therapy in plaque psoriasis management.

## Limitations

Despite the promising findings, our study has several limitations. Firstly, while the IMQ-induced psoriasis-like mouse model is widely used, it primarily represents an acute, Th17-driven inflammatory response and may not fully replicate the chronic and multifactorial nature of human psoriasis. Thus, future work in chronic or longer-term models is warranted. Secondly, the relatively small sample size (n = 5 per group) and use of a single-dose regimen of HMC may restrict the generalizability of the findings, and dose–response relationships should be explored in subsequent studies. Thirdly, the use of female BALB/c mice may limit the extrapolation of results across different strains and sexes, as sex- and strain-related immunological variations can influence disease outcomes. Fourthly, the study relied mainly on cytokine measurements in tissue homogenates; more mechanistic validation through techniques such as NF-κB activation, Western blotting, or TLR7/8 pathway analysis would strengthen the molecular insights. Fifthly, pharmacokinetic and dosing considerations were not addressed, and it remains unclear whether therapeutic plasma levels of HMC achieved in mice are translatable to humans. Sixthly, the potential pharmacological interaction between HMC and methotrexate was not formally assessed by synergy or additivity analyses. Finally, as the data are derived from a single preclinical model, clinical translation remains premature; further preclinical investigations and clinical studies, particularly in plaque-type psoriasis, are required to establish its therapeutic potential.

## Data Availability

The data supporting the findings of this study can be obtained from the corresponding author upon reasonable request.
